# Positive Selection Drives Rapid Evolution of the *meq* Oncogene of Marek’s Disease Virus

**DOI:** 10.1371/journal.pone.0162180

**Published:** 2016-09-23

**Authors:** Abinash Padhi, Mark S. Parcells

**Affiliations:** 1 Department of Animal and Avian Sciences, University of Maryland, College Park, Maryland, 20742, United States of America; 2 Department of Animal and Food Sciences, University of Delaware, 052 Townsend Hall, 531South College Ave, Newark, Delaware, 19716, United States of America; University of Maryland at College Park, UNITED STATES

## Abstract

Marek’s disease (MD), caused by Marek’s disease virus (MDV), a poultry-borne alphaherpesvirus, is a devastating disease of poultry causing an estimated annual loss of one billion dollars to poultry producers, worldwide. Despite decades of control through vaccination, MDV field strains continue to emerge having increased virulence. The evolutionary mechanism driving the emergence of this continuum of strains to increased MDV virulence, however, remains largely enigmatic. Increase in MDV virulence has been associated with specific amino acid changes within the C-terminus domain of Mareks’s EcoRI-Q (*meq*)-encoded oncoprotein. In this study, we sought to determine whether the *meq* gene has evolved adaptively and whether past vaccination efforts have had any significant effect on the reduction or increase of MDV diversity over time. Our analysis suggests that *meq* is estimated to be evolving at a much faster rate than most dsDNA viruses, and is comparable with the evolutionary rate of RNA viruses. Interestingly, most of the polymorphisms in *meq* gene appear to have evolved under positive selection and the time of divergence at the *meq* locus coincides with the period during which the poultry industry had undergone transitions in management practices including the introduction and widespread use of live attenuated vaccines. Our study has revealed that the decades-long use of vaccines did not reduce MDV diversity, but rather had a stimulating effect on the emergence of field strains with increased genetic diversity until the early 2000s. During the years 2004–2005, there was an abrupt decline in the genetic diversity of field isolates followed by a recovery from this bottleneck in the year 2010. Collectively, these data suggest that vaccination seems to not have had any effect on MDV eradication, but rather had a stimulating effect on MDV emergence through adaptation.

## Introduction

Despite the fact that live attenuated vaccines have widely been used to protect host populations against a circulating viral strain, there are several instances where viruses are capable of evolving rapidly in the face of vaccine-elicited protection [1, 2]. As a result, viral strains with new genetic makeup and distinct phenotypic traits have been found to emerge in the population. Mutations in viral proteins that are important to the emergence of the new viral strains with greater fitness are highly unlikely to be non-adaptive or neutral. Knowledge of the viral proteins that have evolved adaptively in vaccinated host populations is therefore crucial for surveillance and control of viral infections.

Marek’s disease (MD), caused by the Marek’s disease virus (MDV), a poultry-borne double-stranded DNA virus, belongs to the family Herpesviridae (subfamily: *Alphaherpesvirinae*; genus: *Mardivirus*), and is one of the unique examples among the known DNA viruses, where new viral strains with greater fitness have continued to emerge in the host population despite widespread vaccination [3–6]. The poultry industries in the United States, China, Australia, India, Japan, the United Kingdom, Poland, and many other European countries [4, 7–12] are reported to be affected by MD and suffer from significant economic loss. Although domestic chickens are the principal host of MDV [4, 13], recent reports indicate the prevalence of this poultry-borne herpesvirus in other wild birds, suggesting cross-species transmission and successful viral adaptation to a wide range of avian host species inhabiting temperate and tropical climates [14, 15]. These findings suggest that MD not only poses a continuing threat to the poultry industry, but also poses a potential concern to wildlife.

MD was initially described by a Hungarian veterinary pathologist, József Marek, in 1907 [16]. Since this time, the clinical features of MD have changed drastically from its initial description [4, 7]. Based on its pathogenicity, MDV is classified into four pathotypes: (1) classical MDV, having mild virulence (mMDVs), which was mostly assumed to have occurred before 1950s, (2) virulent or acute strains (vMDVs) that emerged during the 1950s through the 1960s, the period during which the broiler industry had undergone radical transition to mixed-age farms with a drastic increase in the density of chickens per square meter, (3) very virulent strains (vvMDVs), which emerged during the late 1970s, after the introduction and widespread adoption of the turkey herpesvirus (HVT) vaccine, and the fourth pathotype, designated as very virulent plus (vv+MDVs) emerged after the introduction of the bivalent vaccination (HVT + SB-1) in the early 1990s [4]. After the emergence of vv+MDVs in the US, an attenuated MDV-1 strain, Rispens-CVI988 [17, 18] was introduced in mid-1990s; however, no significant increase in virulence has been noted since this introduction [4, 7]. Despite widespread vaccination over the last four decades, MDV remains as one of the most devastating poultry diseases, worldwide. Despite control through vaccination, the mechanism mediating this evolution of MDV strains to increased virulence remains largely unknown. Interestingly, Schat and Baranowski [19] reported stepwise evolution of virulence of MDV isolates since 1940s till 2000s. According to Schat and Baranowski [19], MDV isolates appeared to be mild (m) during 1940s, virulent (v) during 1960s, very virulent (vv) during 1980s, and vv+ during 2000s. This stepwise increase in virulence seems positively correlated with the introduction of new MDV vaccine each decade. A recent study has also reported an increased pathogenecity of MDV, despite the decades of vaccinations [13]

In the early 2000’s, Shamblin *et al*., [3], reported the association between MDV virulence and patterns of genetic polymorphisms at the C-terminus domain of Mareks’s EcoRI-Q (*meq*)-encoded oncoprotein. Meq is a basic leucine zipper (b-ZIP) protein having characteristics of several viral oncoproteins including v-Jun [20], HBZ of HTLV-I [21], Tat of HIV [22, 23], and EBNA-3C of Epstein- Barr virus (EBV) [24, 25]. Meq is encoded as a 339-amino-acid unspliced open reading frame in vvMDV and vv+ pathotypes of MDV. Mild and virulent MDVs (m/vMDVs) from the 1960s and 1970s encode a larger form of *meq* (398+-amino acids) having multiple duplications of a C-terminal, proline-rich repeat (PRR) domain [3].

Although certain non-synonymous polymorphisms at the *meq*-gene are associated with the MDV virulence [3], it is unclear as to what forces have elicited such evolutionary changes in the vaccinated chicken populations. Given the relatively slow evolutionary rates of the dsDNA viruses [26–28], the *meq* gene, would be expected to evolve at a similar rate. Alternatively, most of these polymorphisms are likely to be adaptive and have evolved under strong, positive selection pressure. In such circumstance, the mutation rate of the *meq* gene would be expected to be significantly higher than the usual rate that has been reported for dsDNA viruses. Given the ongoing risk posed by MDV, it is crucial to estimate the rates of evolution and the pervasive role of positive selective pressures on MD viral emergence. Here, we sought to determine whether the *meq* gene has evolved adaptively and whether past vaccination efforts have had any significant effect on the reduction of MD viral diversity over time.

By performing a Bayesian coalescent analysis [29–31] using serially sampled *meq*-gene sequence data and site-specific selection analyses [32], we report the evolutionary parameters and gain valuable insight into the epidemiology of this highly contagious poultry herpesvirus, as well as the underlying genetic mechanisms that maintain polymorphisms in the *meq* gene. Understanding the past population dynamics and determining the evolutionary rate of change for *meq* may provide important insight into the vaccination, management and factors driving the selection for and fixation of particular mutations.

## Materials and Methods

### Phylogenetic analyses

A total of 84 *meq* gene complete nucleotide coding sequences, including 44 sequences of known MDV pathotypes that were previously reported by several authors [3, 4, 8–10, 12, 15] were retrieved from GenBank (S1 Table). Most of these sequences were from the flocks that were vaccinated either with HVT, bivalent vaccine (HVT + SB-1), Rispens-CVI988, or by 814-based-vaccines [3, 4, 9, 10, 12]. Sequences were aligned using MEGA, version 4 [33]. To infer the phylogenetic relationships among the known pathotypes, maximum likelihood (ML) tree under the appropriate nucleotide substitution model was reconstructed using PhYML, version 3 [34], and nodal supports were estimated with 1000 bootstrap replicates. To assess the robustness of ML tree topologies, we generated posterior probabilities for each node by performing BMCMC (Bayesian Markov Chain Monte Carlo) analyses implemented in MrBayes, version 3.1.2 [35].

### Estimation of evolutionary parameters and population dynamics

We evaluated the clock-like behavior of the *meq* gene sequence data set (n = 69) using a conservative assessment of root-to-tip genetic distance regression analyses implemented in Path-O-Gen ver. 1.3 (http://tree.bio.ed.ac.uk/software/pathogen/; [36]). The coefficient of correlation was determined by fitting a regression of the year-of-sampling against the root-to-tip genetic distance of each sample, measured from an ML tree. We then used a Bayesian Markov Chain Monte Carlo (MCMC) approach to estimate the overall substitution rate (measured in substitutions per site per year) for the *meq* gene under the strict (constant molecular clock) and relaxed (uncorrelated lognormal) molecular clocks [29, 31] implemented in BEAST version, 1.7.1 [30].

To estimate the evolutionary rates and infer the population dynamics, we used all the complete nucleotide coding sequences of *meq* gene (n = 69) whose year of isolation were available. The MCMC chains were run for sufficient time to achieve convergence. Phylogenies were evaluated using a chain length of 40 million states under a General Time Reversible (GTR) model with proportion of invariable sites (I) and gamma distribution shape parameter (G). Uncertainty in the data was described by the 95% high-posterior density (HPD) intervals. Convergence of trees was checked using Tracer ver. 1.5 (available at: http://beast.bio.ed.ac.uk/Tracer). The inferred trees were visualized using FigTree ver. 1.3.1 (available at: http://tree.bio.ed.ac.uk/software/figtree/). We utilized the Bayesian skyline plot (BSP) [31] as a coalescent prior to inferring the population history of MDV. The BSP estimates changes in effective population size of MDV through time, measured in terms of relative genetic diversity. The best-fit clock model was evaluated from the coefficient of variation (CoV) values. The Maximum Clade Credibility (MCC) tree was generated using the TreeAnnotator software program implemented in the BEAST package.

### Tests for positive selection

Prior to selection analyses, a recombination detection program (RDP) implemented in the RDP3 software package [37] was used to determine if any of the *meq* genes showed evidence of recombination. The analyses revealed no evidence of recombination, thus allowing us to test for positive selection. We performed ML-based selection analysis to determine whether the *meq* gene is evolving under positive selection. Several codon-specific models (M1a, M2a, M7, M8, and M8a) implemented in the codeml program of PAML [32] were used to determine which residues are evolving under positive selection. Sites models allow the rate of nonsynonymous (dN) to the synonymous (dS) ratio (dN/dS = ω) to vary among residues. The input trees for selection analyses were reconstructed using the PhyML program. The likelihood ratio test (LRT’s) was used to compare M1a, M7, and M8a models that assume no positive selection (ω < 1) with the M2a and M8 models that assume positive selection (ω > 1). Sites with Bayes Emperical Bayes (BEB) posterior probability ≥ 0.95 were considered to be evolved under strong positive selection. Positively selected sites were also detected using random effect likelihood (REL) and Fast Unbiased Bayesian Approximation (FUBAR) methods [38–40] via the Datamonkey website [39]. Bayes factor greater than 50 was used as thresholds for strong evidence of selection in REL.

## Results and Discussion

### Phylogenetic relationships of the pathotypes with vaccine strains

**Fig 1:**
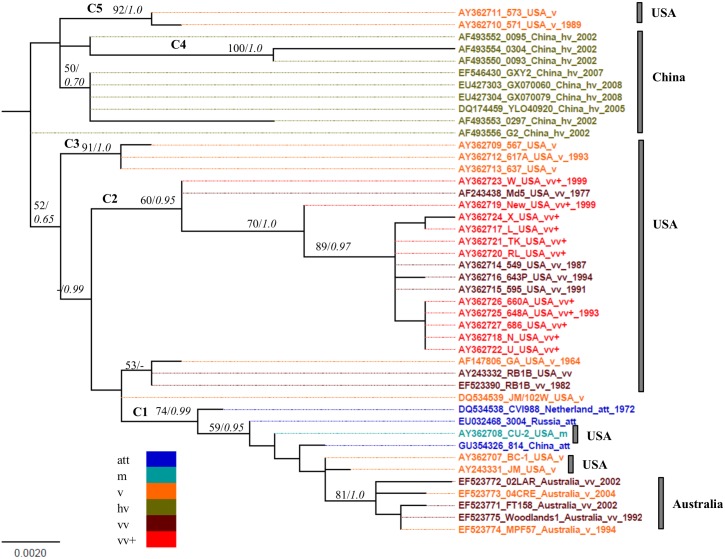
Maximum likelihood tree of MDV *meq* sequences. Maximum likelihood tree inferred from *meq* gene complete nucleotide sequence data of 44 MDV isolates with known pathotypes are shown. Bars at right identify the phylogenetic clustering of pathotypes from different geographic regions. Bootstrap supports/posterior probabilities are mentioned at the base of the nodes. Nodes with high bootstraps/posterior probabilities (> 60/0.95) are indicated by clusters, C1-C5. Abbreviations: m: mild, att: attenuated, hv: high virulence, vv: very virulence, v: virulent, vv+: very virulent plus.

Phylogenetic relationships among the 44 pathotyped MDV strains, including three vaccines were inferred from the *meq* gene sequences, using ML and Bayesian MCMC approaches with bootstrapping and posterior probability analyses to assess clade robustness (Fig 1). Five clusters (C1 –C5) were defined with bootstrap supports ≥ 60 and posterior probability > 0.9 (Fig 1). MDV pathotypes from the USA appear to be on the multiple clusters (i.e. C1, C2, C3, and C5), whereas cluster 5 (C5) is represented by samples from China. All the five viral strains from Australia, along with a few strains from the USA, tended to cluster with the three vaccine strains, whereas all the vv+MDV pathotypes from the USA that were sampled between the years 1987–1995 formed a unique cluster (Fig 1). Although most of the viral samples with known pathotypes from China seem to form a cluster, the cluster is not strongly supported (Fig 1). Nevertheless, our analyses have revealed that all the viral strains with known pathotypes are not phylogenetically closely related to the vaccine strains.

### Evolutionary rates and past population dynamics

**Fig 2:**
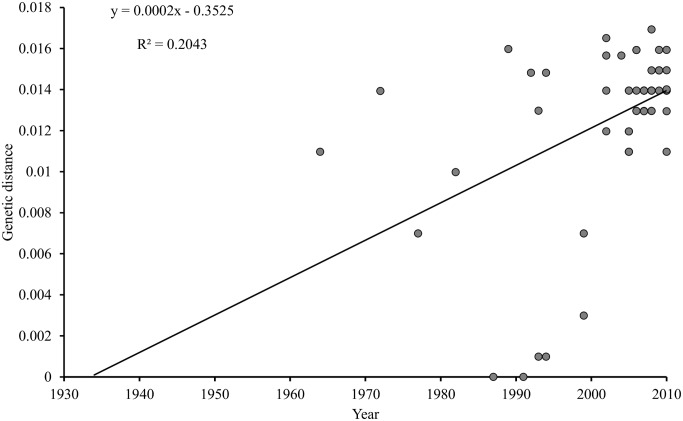
The root-to-tip genetic distance based on *meq* gene versus year of MDV isolation. The regression coefficient (R^2^) estimates the fit of the data to a strict molecular clock by testing the degree of influence sampling time has over the amount of pairwise diversity in the data. This analysis suggests the presence of temporal structure for *meq* gene of MDV.

**Fig 3:**
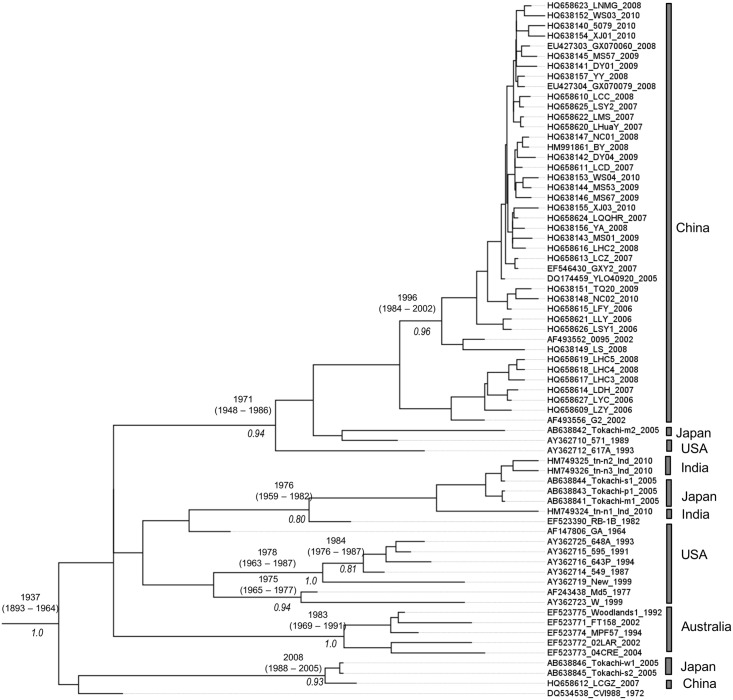
Maximum Clade Credibility (MCC) tree inferred from the Bayesian analysis of the MDV *meq* gene sequences. The mean TMRCAs with confident intervals (above the nodes) and the posterior probabilities (below the nodes) are mentioned.

**Fig 4:**
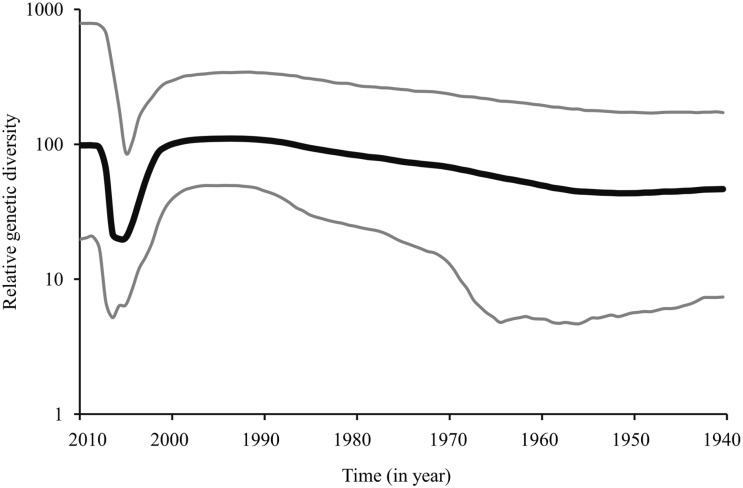
Bayesian skyline plot (BSP) inferred from the *meq* gene sequences. The BSP above depicts the relative genetic diversity of MDV over time. The plot depicting MDV population had recovered from a recent bottleneck (~2005–2008).

The clock-like behavior of the *meq* gene was assessed by plotting the root-to-tip genetic distance from a ML tree against the sampling-year of each MDV isolate. The resultant coefficient of correlation (R^2^) weakly suggested the presence of clock-like evolution of the *meq* gene in MDV (Fig 2). Although weak (i.e., R^2^ = 0.204), yet the dataset exhibits a positive correlation between genetic divergence and sampling time, thus indicating an extent of temporal structure in the data, and therefore, warrants further molecular clock-based phylogenetic analysis. We estimated the evolutionary parameters under strict and relaxed clock models implemented in the BEAST program [29, 30]. We inferred the evolutionary rates and past population dynamics of MDV using a Bayesian coalescent approach [29, 31]. This analysis was based on all the complete *meq* gene sequences (n = 69) in the dataset that had a date of isolation. Under the relaxed clock model, although higher mean CoV value was observed for *meq*, the lower 95% HPD is close to zero, indicating that the strict molecular clock could not be rejected (Table 1). The mean rate and TMRCAs estimated by both clocks are comparable and showed a narrow range of posterior distributions around the estimated mean posterior values in respective clock models (Table 1). The mean evolutionary rate for *meq* is estimated to be 1.02 (95% HPD: 0.50–1.60) ×10^−4^ substitutions/site/year and the estimated mean TMRCA is approximately around the year 1935 (95% HPD: 1893–1964) (Table 1). Most of the internal nodes were estimated to have diverged during the period of 1950s to early 2000s (Fig 3). Bayesian skyline plot (BSP) inferred from the *meq* gene sequence data was used to estimate the genetic diversity over time (Fig 4). The genetic diversity appeared to be relatively constant until the late-1950s, followed by a steady increase until early-2000. During 2004–2005, there was an abrupt decline in the genetic diversity followed by the recovery from this bottleneck in 2010 (Fig 4).

**Table 1:** Bayesian estimates of the evolutionary rate and TMRCAs (in year) inferred from the MDV *meq* gene.

N	Year range	Clock Model	Mean substitution rate (95% HPD)	TMRCA (95% HPD)	CoV (95% HPD)
			(in × 10^−4^ subs/site/ year)	(in year)	
69	1964–2010	Strict	0.90 (0.50–1.38)	1934 (1896–1959)	NA
		Relaxed	1.02 (0.55–1.60)	1937 (1893–1964)	0.58 (0.000089–1.36)

N: Number of sequences.

HPD: Highest Posterior Density.

TMRCA: Time to the Most Recent Common Ancestor.

NA: Not Applicable.

CoV: Coefficient of Variation.

### Tests for positive selection

**Fig 5:**
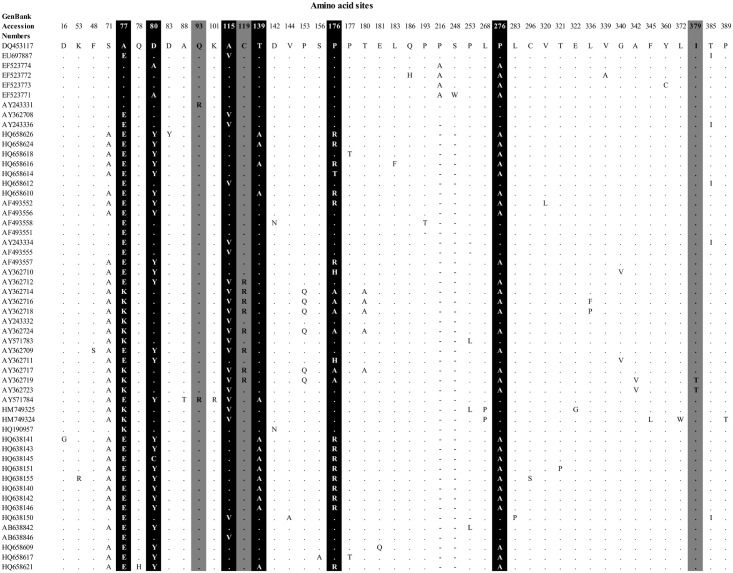
Variable amino acid sites in *meq* genes. Positively selected with posterior probability > 0.95 and sites with posterior probability between 0.90–0.95 are highlighted in black and grey colors, respectively.

To determine the importance of natural selection in the evolution and divergence of *meq*, we performed codon-specific selection analyses using several models implemented in PAML program [32, 41] and FUBAR, REL methods implemented in the Datamonkey web server [39]. We performed the analyses on two datasets: (1) all the datasets and (2) exclusively on the known pathotypes. For both datasets, the overall dN/dS for *meq* is greater than one (Table 2), indicating most of the polymorphisms in *meq* have evolved under strong positive selection. To know which residues in *meq* have evolved under positive selection pressure, we have performed codon-by-codon selection analyses. For both datasets, the null models that assume neutral evolution were rejected (p < 0.00001) by the likelihood ratio tests (LRTs) (Table 2), thus indicating the pervasive role of positive selection in the evolution and divergence of *meq* gene in MDV. Notably, codons -77, 80, 115, 119, 139, 176, 276, 379, and 385 in both datasets have evolved under strong positive selection with BEB > 0.90 (Table 2; Fig 5). Most of these sites were detected to be under positive selection by FUBAR and REL methods (Table 3). We also performed selection analyses on gycoprotein B (gB); however, none of the sites were detected to be under positive selection. In contrast to *meq*, gB is appeared to be highly conserved. Amino acid alignments of gB8 are shown in S1 Fig.

**Table 2:** Sites under positive selection detected by PAML methods. Likelihood ratio tests for positive selection for the MDV *meq* gene and positively selected sites detected by M2a and M8 selection model.

Group	Overall dN/dS	Model Comparison	2*Δl*	df	*p*-value	Postively-selected sites^a^
All	1.566	M1a vs M2a	42.623	2	7.464 x 10^−8^	**77**, **80**, 93, **115**, 119, **139**, **176**, **276**, 342, 379
		M7 vs M8	42.621	2	1.936 x 10^−8^	**77**,**80**, **93**, **119, 115**, **139**, **176**, 253, 268, **276**, 336, 342, 379, 385
		M8 vs M8a	38.307	1	1.010 x 10^−8^	
Pathotype	1.835	M1a vs M2a	35.589	2	1.869 x 10^−8^	**77, 80, 115, 119**, 139, **176**, **276**, **303**, 336, 342, **379**, 385
		M7 vs M8	35.589	2	1.869 x 10^−8^	**77, 80, 115, 119**, **139**, **176**, **276**, **303**, **336**, **342**, **379**, **385**
		M8 vs M8a	34.045	1	5.385 x 10^−9^	

dN/dS: Rate of nonsynonymous (dN) to the synonymous (dS) substitutions.

Null model (Neutral): M1a, M7, M8a.

Alternative model (Selection): M2a, M8.

*Δl*: Difference between the likelihood scores.

df: Degrees of freedom.

^a^: Sites with posterior probability > 0.95 are in bold, > 0.90 and < 0.95 underlined.

**Table 3:** Positively selected sites are shown as detected by REL and FUBAR methods.

REL		FUBAR	
Codon	Posterior Probability	Codon	Posterior Probability
77	0.981	77	0.9520
80	0.986	80	0.9640
93	0.915	93	0.9159
115	0.989	115	0.9765
139	0.914	139	0.9377
176	0.992	176	0.9873
253	0.990	253	0.9816
276	0.993	276	0.9879
336	0.892	336	0.9247
342	0.916	342	0.9323
385	0.910	385	0.9322

REL: Random Effects Likelihood.

FUBAR: Fast Unconstrained Bayesian AppRoximation.

Understanding the evolutionary basis of viral emergence is crucial for effective vaccine development. Despite decades of vaccination efforts, the continuum of increasing virulence of MDV has been a serious concern [4, 13, 19, 42, 43]. The increased genetic polymorphisms in *meq* together with the reports of the association between MDV virulence and specific nonsynonymus mutations at the C-terminus domain of *meq* indicate the putative role of *meq* in the evolution of MDV virulence [3, 12]. In this study, we report that (1) *meq* is evolving at a much faster rate than most dsDNA viruses, (2) most of the polymorphisms in the *meq* gene have evolved under positive selection, (3) the time of divergence at the *meq* locus coincides with the period during which the poultry industry had undergone transitions in management practices including the introduction and widespread use of live attenuated vaccines, and (4) the decades-long use of vaccines did not reduce MDV diversity, but rather had a stimulating effect on the emergence of field strains with increased genetic diversity.

Most of the dsDNA viruses, including herpsviruses, have evolved at slower rates, mostly within a range of 10^−7^ to 10^−5^ substitutions per site per year [26, 28, 44–49]. In contrast, the *meq* gene of MDV is evolving at a rate that is comparable with the rates of RNA viruses [50]. Although several factors could be associated with the extremely slow evolutionary rate of most dsDNA viruses [26, 28, 49, 51], the assumption of host-pathogen co-divergence, where most of the dsDNA viruses are thought to evolve with their respective hosts, is a plausible explanation for this observation [27]. Utilizing the gene sequence data within a Bayesian framework, and without considering the assumption of host-virus co-divergence, Fitch *et al*., [27] reported a large-scale variation in the substitution rate of dsDNA viruses (in the order of 10^−3^ to 10^−6^ substitutions/site/year) and interestingly, substitution rates of some of the dsDNA viruses are comparable with that of RNA viruses. Although substitution rates of dsDNA viruses are likely to be gene-specific, genes that have evolved under positive selection have elevated substitution rates [27]. To determine the putative role of positive selection in the evolution and divergence of the MDV *meq* gene, we performed the ML-based positive selection analyses. The overall dN/dS for *meq* was 1.54, indicating the evidence of adaptive evolution of *meq* driven by positive selection. Thus, positive selection is the most likely contributing factor for such inflated substitution rate of *meq*.

From the results of our present study, although it is apparent that positive selection appears to be a cause of the rapid evolution of the *meq* gene of MDV, it is unclear as to what factors drive this gene to be under positive selection. Positive selection largely reflects the viral adaptation against host immune defenses, with adaptive changes providing beneficial consequences for the virus to evade the host’s acquired/intrinsic immunity. Although vaccines may provide protection against the viruses, viruses have the ability to adapt rapidly to the changing environment by modification of key amino acid residues in viral proteins that are involved in the host-pathogen interaction process. In the case of *meq*, however, it is unclear what adaptive advantage these substitutions would confer and why in particular they would be selected through the use of vaccines that lack a *meq* homolog (HVT, SB-1).

Nevertheless, such adaptive changes through positive selection pressures (i.e., accumulation of an excess of nonsynonymous changes relative to synonymous changes over time), may cause the emergence of new viral strains with greater fitness in the vaccinated populations. Under these circumstances, birds vaccinated with the old vaccines are unlikely to overcome the re-infection with the new viral strains. One might also expect the existence of multiple viral lineages of MDV with distinct genetic fitness. Human influenza A viruses are the classic example of such adaptation through the rapid evolutionary changes at antigenic sites (HA, NA). Because of this adaptation, vaccines for influenza viruses are seasonal and require continuous monitoring, surveillance, and determination of the pattern of genetic polymorphisms at the antigenic sites for the effective design of vaccines [1, 2]. This does not appear to be the case for MDV-1 strains and *meq*, as *meq* is a nuclear/nucleolar protein, and is not encoded by HVT and MDV-2 (SB-1) vaccine strains. Additionally, prior work has shown that the major structural genes of MDV-1 field strains do not appear to be under the level of selection that *meq* appears to be [52, 53].

Several mutations at the C-terminus region of *meq* gene (codons-80, 115, 176, 276) that were previously reported to be associated with MDV virulence [3] have apparently evolved under positive selection (see Fig 5). Recently, Australian isolates were found to increase in virulence with particular mutations in the tandem direct repeats of proline residues [54]. The model that they put forth was that the fewer prolines present in the C-terminus of *meq*, the greater the level of virulence. These adaptive evolutionary changes at the *meq* oncogene are likely to have some functional consequences and MDV evolution. Therefore, further experiments on the putative role of these specific mutations would provide a better understanding on the MD viral emergence and genetic basis of its adaptation. A detailed understanding on the geographic dominance of different MD viral strains is also required for effective vaccine development. Biochemically, changes in *meq* have been associated with increased transcriptional activation [55, 56], increased invasiveness of cells expressing *meq* isoforms, and most recently, changes in tumor composition [57]. How these particular mutations directly contribute to MDV changes in virulence await further mutant construction and characterization.

Prior to the 1950s, MDV was less virulent; however, the virus became a concern after the introduction of vaccines and significant changes in brooding practices [4]. Such changes might have a significant impact on the viral diversity, and the virus may have evolved much faster rate through adaptation. The *meq* gene based Bayesian analyses have revealed that the MDV is estimated to have originated sometime between the year 1893–1964; however, a rapid divergence of MDV leads to multiple lineages arising between 1950 to the early 2000s. Despite vaccination efforts, the Bayesian skyline plots revealed a steady increase in viral diversity from the 1950s until the mid-2000s. This pattern clearly indicates that vaccination did not reduce viral diversity, but increased it. The sharp decline in viral diversity during the early 2000s could possibly be the effect of the introduction of Rispens (CVI988) in the US and 814-vaccines in China. However, the recovery of the diversity of viral populations suggests vaccine failure.

## Conclusions

Collectively, although our analyses revealed an interesting evolutionary dynamics of MDV, it is possible that the geographic origin of the viral strains may have profound influence on the rates of evolution and population dynamics of MDV. However, due to limited sample size, we could not perform the geographic-based analyses. Therefore, upon availability of large cohorts of samples representing different geographic regions, future study can be carried out to unravel the phylogeographic structure, distinct selection profiles, as well as to know whether there are significant differences in evolutionary rates among the geographic regions. Additionally, future studies should also focus on the evolutionary dynamics of MDV using other genes such as oncogenicity-associated phosphoproteins pp38 and pp24, lipase homologue, a CxC chemokine, and unique proteins of unknown function MDV087 and MDV097 and MDV093 that contribute towards virulence of MDV.

## Supporting Information

S1 Fig Amino acid alignment of Eight Glycoprotein B sequences of MDV. Amino variable sites are highlighted in gray color. Those strains having identical sequence are:
AY510475 is Identical to: AF243438, KT833851, and KT833852. JX844666 is Identical to: EU499381, JQ809692, JQ836662, JQ809691, EF523390, U39846, DQ530348, JQ806362, JQ806361, AF147806, JQ820250, JF742597, AY129966, and D13713.
(DOCX)

S1 Table GenBank accession number, name of the isolate, country of origin, and year of isolation of MDV isolates analyzed in the present study. (DOCX)

S2 Table Likelihood ratio statistics for positive selection for glycoprotein B (gB) of MDV. (DOCX)
