# Supplementary material for: Positive Selection Drives Rapid Evolution of the meq Oncogene of Marek’s Disease Virus
Source: PLoS One. 2016 Sep 23;11(9):e0162180. doi: 10.1371/journal.pone.0162180 (PMC5035050; doi:10.1371/journal.pone.0162180)
Supplement: S1 Fig — Amino variable sites are highlighted in gray color. Those strains having identical sequence are: AY510475 is Identical to: AF243438, KT833851, and KT833852. JX844666 is Identical to: EU499381, JQ809692, JQ836662, JQ809691, EF523390, U39846, DQ530348, JQ806362, JQ806361, AF147806, JQ820250, JF742597, AY129966, and D13713. (DOCX) [file pone.0162180.s001.docx]

**Figure S1.** Amino acid alignment of the 8 (only unique sequences are shown in the alignment) Glycoprotein B sequences of MDV. Amino variable sites are highlighted in gray color.

Identical sequence list:

AY510475 is Identical to: AF243438, KT833851, and KT833852.

JX844666 is Identical to: EU499381, JQ809692, JQ836662, JQ809691, EF523390, U39846, DQ530348, JQ806362, JQ806361, AF147806, JQ820250, JF742597, AY129966, and D13713.
