# Supplementary material for: Positive Selection Drives Rapid Evolution of the meq Oncogene of Marek’s Disease Virus
Source: PLoS One. 2016 Sep 23;11(9):e0162180. doi: 10.1371/journal.pone.0162180 (PMC5035050; doi:10.1371/journal.pone.0162180)
Supplement: S1 Table — (DOCX) [file pone.0162180.s002.docx]

**Supplementary Tables**

**Table S1.** GenBank accession number, name of the isolate, country of origin, and year of isolation of MDV isolates analyzed in the present study.

|  |  |  |  |  |
| --- | --- | --- | --- | --- |
|  | Accession number | Isolate | Country of Origin | Year of Isolation |
|  | AB638841 | Tokachi-m1 | Japan | 2005 |
|  | AB638842 | Tokachi-m2 | Japan | 2005 |
|  | AB638843 | Tokachi-p1 | Japan | 2005 |
|  | AB638844 | Tokachi-s1 | Japan | 2005 |
|  | AB638845 | Tokachi-s2 | Japan | 2005 |
|  | AB638846 | Tokachi-w1 | Japan | 2005 |
|  | AF147806 | GA | USA | 1964 |
|  | AF243438 | Md5 | USA | 1977 |
|  | AF493552 | 95 | USA | 2002 |
|  | AF493556 | G2 | USA | 2002 |
|  | AY243331 | JM | USA | - |
|  | AY362707 | BC-1 | USA | - |
|  | AY362708 | CU-2 | USA | - |
|  | AY362709 | 567 | USA | - |
|  | AY362710 | 571 | USA | 1989 |
|  | AY362711 | 573 | USA | - |
|  | AY362712 | 617A | USA | 1993 |
|  | AY362713 | 637 | USA | - |
|  | AY362714 | 549 | USA | 1987 |
|  | AY362715 | 595 | USA | 1991 |
|  | AY362716 | 643P | USA | 1994 |
|  | AY362717 | L | USA | - |
|  | AY362718 | N | USA | - |
|  | AY362719 | New | USA | 1999 |
|  | AY362720 | RL | USA | - |
|  | AY362721 | TK | USA | - |
|  | AY362722 | U | USA | - |
|  | AY362723 | W | USA | 1999 |
|  | AY362724 | X | USA | - |
|  | AY362725 | 648A | USA | 1993 |
|  | AY362726 | 660A | USA | - |
|  | AY362727 | 686 | USA | - |
|  | DQ174459 | YLO40920 | China | 2005 |
|  | DQ534538 | CVI988 | Netherlands | 1972 |
|  | DQ534539 | JM/102W | USA | - |
|  | EF523390 | RB1B | USA | 1982 |
|  | EF523771 | FT158 | Australia | 2002 |
|  | EF523772 | 02LAR | Australia | 2002 |
|  | EF523773 | 04CRE | Australia | 2004 |
|  | EF523774 | MPF57 | Australia | 1994 |
|  | EF523775 | Woodlands1 | Australia | 1992 |
|  | EF546430 | GXY2 | China | 2007 |
|  | EU427303 | GX070060 | China | 2008 |
|  | EU427304 | GX070079 | China | 2008 |
|  | HM749324 | tn-n1 | India | 2010 |
|  | HM749325 | tn-n2 | India | 2010 |
|  | HM749326 | tn-n3 | India | 2010 |
|  | HM991861 | BY | China | 2008 |
|  | HQ638140 | 5079 | China | 2010 |
|  | HQ638141 | DY01 | China | 2009 |
|  | HQ638142 | DY04 | China | 2009 |
|  | HQ638143 | MS01 | China | 2009 |
|  | HQ638144 | MS53 | China | 2009 |
|  | HQ638145 | MS57 | China | 2009 |
|  | HQ638146 | MS67 | China | 2009 |
|  | HQ638147 | NC01 | China | 2008 |
|  | HQ638148 | NC02 | China | 2010 |
|  | HQ638149 | LS | China | 2008 |
|  | HQ638151 | TQ20 | China | 2009 |
|  | HQ638152 | WS03 | China | 2010 |
|  | HQ638153 | WS04 | China | 2010 |
|  | HQ638154 | XJ01 | China | 2010 |
|  | HQ638155 | XJ03 | China | 2010 |
|  | HQ638156 | YA | China | 2008 |
|  | HQ638157 | YY | China | 2008 |
|  | HQ658609 | LZY | China | 2006 |
|  | HQ658610 | LCC | China | 2008 |
|  | HQ658611 | LCD | China | 2007 |
|  | HQ658612 | LCGZ | China | 2007 |
|  | HQ658613 | LCZ | China | 2007 |
|  | HQ658614 | LDH | China | 2007 |
|  | HQ658615 | LFY | China | 2006 |
|  | HQ658616 | LHC2 | China | 2008 |
|  | HQ658617 | LHC3 | China | 2008 |
|  | HQ658618 | LHC4 | China | 2008 |
|  | HQ658619 | LHC5 | China | 2008 |
|  | HQ658620 | LHuaY | China | 2007 |
|  | HQ658621 | LLY | China | 2006 |
|  | HQ658622 | LMS | China | 2007 |
|  | HQ658623 | LNMG | China | 2008 |
|  | HQ658624 | LQQHR | China | 2007 |
|  | HQ658625 | LSY2 | China | 2007 |
|  | HQ658626 | LSY1 | China | 2006 |
|  | HQ658627 | LYC | China | 2006 |
|  |  |  |  |  |
