# Supplementary material for: Positive Selection Drives Rapid Evolution of the meq Oncogene of Marek’s Disease Virus
Source: PLoS One. 2016 Sep 23;11(9):e0162180. doi: 10.1371/journal.pone.0162180 (PMC5035050; doi:10.1371/journal.pone.0162180)
Supplement: S2 Table — (DOCX) [file pone.0162180.s003.docx]

**Table S2. Likelihood ratio statistics for positive selection for glycoprotein B (gB) of MDV.**

| Model Comparison | *2*Δ*l* | df | *p* | Positvely selected sites |
| --- | --- | --- | --- | --- |
| M1a vs M2a | 2E-06 | 2 | 1.000 | None |
| M7 vs M8 | 1E-05 | 2 | 1.000 | None |
| M8 vs M8a | 1.2E-05 | 1 | 0.997 | None |
| Neutral model: M1a, M7, M8a; Selection model: M2a, M8 | | | | |
